# Impact of the COVID-19 pandemic on emergency department visits for genitourinary trauma

**DOI:** 10.1186/s12894-022-01041-4

**Published:** 2022-06-15

**Authors:** Behnam Nabavizadeh, Nizar Hakam, Behzad Abbasi, Nathan M. Shaw, Benjamin N. Breyer

**Affiliations:** 1 Department of Urology, University of California San Francisco, San Francisco, CA USA; 2 Department of Biostatistics and Epidemiology, University of California San Francisco, San Francisco, CA USA; 3 Zuckerberg San Francisco General Hospital and Trauma Center, 1001 Potrero Suite 3A, San Francisco, CA 94110 USA

**Keywords:** Genitourinary, Trauma, COVID-19, Pandemic

## Abstract

**Introduction:**

The mean number of emergency department visits for all-cause traumas has declined significantly during the COVID-19 pandemic. We aim to identify how a global pandemic and social distancing could affect the trends and pattern of genitourinary traumas.

**Methods:**

We queried the National Electronic Injury Surveillance System to obtain consumer product-related genitourinary injuries leading to emergency department visits. Using three key events in 2020, we divided the study period to three intervals: January 20, when the first COVID-19 case was confirmed in the United States; March 13, when a national state of emergency was declared; April 20, when Texas became the first state to start a phased reopening of economy. We compared the injury characteristics in 2020 to their identical intervals in 2019.

**Results:**

Daily emergency department visits dropped significantly during the national lockdown (mean 131.5 vs. 78; Δ-40.7%; *p* < 0.01). The genitourinary injuries decreased significantly in children ≤ 17 years (*p* < 0.01), males (*p* < 0.001), and White population (*p* < 0.01). However, it did not change significantly in adults 18–64 years (*p* = 0.92), old adults ≥ 65 years (*p* = 0.37), females (*p* = 0.60), Black population (*p* = 0.90), other/unknown races (*p* = 0.93), and for injuries sustained at home (*p* = 0.75) and public (*p* = 0.11) locations. During the lockdown period, injuries associated with toilets/toilet seats (− 320, − 74.6%), day wear (− 266, − 77.7%), beds/bedframes (− 209, − 64.2%) decreased while injuries associated with knickknacks/statues/vases (+ 154, n/a), sofas/couches/divans (+ 130, 2,684%), and razors/shavers (+ 99, n/a) increased.

**Conclusions:**

The COVID-19 lockdown had a significant impact on genitourinary traumas. The contributing factors could be investigated further to prevent such injuries during deconfinement periods.

## Introduction

On January 20, 2020, the Centers for Disease Control and Prevention (CDC) confirmed the first case of coronavirus disease 2019 (COVID-19) in the United States (US) which was followed by a national state of emergency declared on March 13, 2020 [1]. In order to impede the spread of the disease, authorities imposed major restrictions on social activities, public gatherings, and travels leading to school closures and stay-at-home orders [2]. As a result, normal daily life changed dramatically.

The COVID-19 pandemic was associated with an unprecedented surge in the number of patients referring to hospitals which has placed an unexpected burden on health systems necessitating reallocation of hospital resources [3]. To tackle the shortcomings stemmed from the influx of patients with COVID-19 to emergency departments (ED) and hospitals, health authorities have introduced several regulations, including the promotion of telehealth to confine nonessential ED/hospital visits and suspension of nonemergent elective services. Likewise, urologic care has been affected by the regulations and efforts to redistribute the resources [4–6].

As reported by CDC, the mean number of all-cause ED admissions during the COVID-19 pandemic has declined by 42% after the declaration of a national state of emergency for COVID-19 [7]. Several studies have addressed the impact of the ongoing pandemic on trauma, indicating a decrease in trauma ED visits [8]. However, such evidence on the trends of genitourinary trauma during the COVID-19 pandemic is still lacking. Using a nationally representative database of ED visits, we aim to present a detailed insight on the impact of the COVID-19 pandemic on the trends of genitourinary injuries. We hypothesize that the average daily ED visits as a result of genitourinary traumas decreased significantly after the declaration of a national state of emergency.

## Methods

### Database

We queried the National Electronic Injury Surveillance System (NEISS) database to obtain injuries leading to ED visits. This database is operated by the US Consumer Product Safety Commission and collects data on consumer product-related injuries occurring in the US from approximately 100 EDs selected as a probability sample of all US hospitals with EDs [9]. Therefore, this data can be used to produce national estimates of the number of injuries associated with specific consumer products. The NEISS data is deidentified and publicly available, therefore this study was deemed exempt from obtaining institutional review board approval.

### Study population and COVID-19 timeline

We used the NEISS Query Builder to obtain all injuries to pubic region (code 38) and lower trunk (code 79) occurred in 2019 and 2020. Using genitourinary-related keywords, we reviewed all case narratives to find injuries to different genitourinary organs: kidney (keywords: kidney, renal), ureter (keyword: ureter), bladder (keyword: bladder), urethra (keyword: urethra), penis (keywords: penis, penile), scrotum/testis (keywords: scrotum, scrotal, testis, testicular), female external genitalia (vagina, vulva, labia, fourchette), and other (genital, genitourinary, perineum, GU). Then, we reviewed all narratives to code the injured body part variable.

The study period was divided into three intervals based on the timeline of COVID-19 response in the US. The key events included January 20, 2020, when the CDC confirmed the first COVID-19 case in the US; March 13, 2020, when a national state of emergency was declared in the US; April 20, 2020, when Texas became the first state to start a phased reopening of economy.

### Data analysis

We used sample weights to produce national estimates of genitourinary injuries. We compared the injury characteristics in 2020 to their equivalent intervals in 2019. We applied Pearson's chi-square, Wilcoxon Rank Sum, and t-tests as appropriate to determine the differences between the pandemic and 2019 control periods. All *p* values were two-sided and considered to be statistically significant if < 0.05. All statistical tests were performed using IBM SPSS Statistics, version 24 (IBM Corp., Armonk, NY, US). The STROBE (Strengthening the Reporting of Observational Studies in Epidemiology) statement was followed for the design and reporting of this study [10].

## Results

**Fig. 1:**
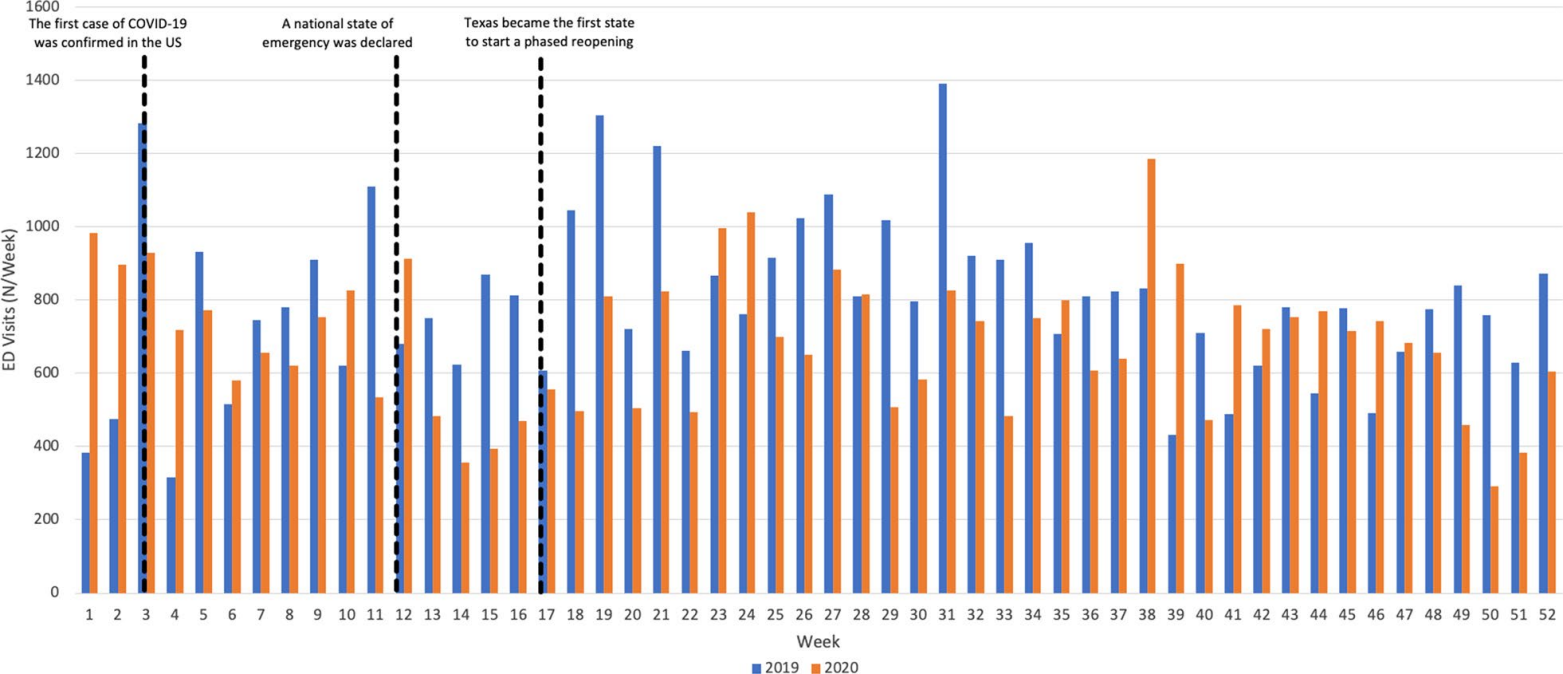
Weekly emergency department visits for genitourinary traumas associated with consumer products in 2019 and 2020 in the United States

A total of 1,419 and 1,344 actual cases corresponding to 41,427 (95%CI 32,771–50,084) and 35,861 (95%CI 27,529–44,193) weighted cases of genitourinary trauma presented to the US EDs in 2019 and 2020, respectively. Daily ED visits dropped significantly during the national lockdown (from March 13, 2020 to April 19, 2020) compared to the similar interval in 2019 (mean 131.5 vs. 78; Δ-40.7%; *p* < 0.01; Fig. 1). However, no significant difference existed during the first (from January 20 to March 12; mean 105.7 vs. 104.3; Δ-1.3%; *p* = 0.61) and the third intervals (after April 20; mean 118.6 vs. 103.7; Δ-12.6% *p* = 0.05; Fig. 1). The mean age of patients presenting with genitourinary injuries was higher during the lockdown period (27.3 years, standard error [SE] 4.3) as compared to the control period (21.2 years, SE 2.4); however, this increase was not statistically significant (*p* = 0.18).

Table 1 shows the patient and injury characteristics stratified by the lockdown and 2019 control periods. The distributions of several variables were significantly different between the two periods including sex (*p* = 0.04), race (*p* = 0.03), diagnosis (*p* = 0.04), and location of injury (*p* = 0.03). However, age groups (*p* = 0.33), primary genitourinary organs injured (*p* = 0.10), and final disposition of the patients (*p* = 0.19) remained proportionally unchanged. Penile injuries were the most common type of injuries in 2019, however it became the third on the list in 2020. There were 121 weighted cases with simultaneous injury to another genitourinary organ (5 actual cases, all in 2019).

**Table 1:** Comparison of patient/injury characteristics between the lockdown and control periods (from March 13 to April 19)

	Control period (2019)	Lockdown period (2020)	*P* value
Total number, estimates (unweighted n; 95%CI)	4,601 (158; 3,169–6,039)	2,731 (97; 1,855–3,608)	**0.01**
Age, mean (SE)	21.2 (2.4)	27.3 (4.3)	0.18
Age groups, n (95%CI, %)			
Children (0–17 years) Adults (18–64 years) Old adults (≥ 65 years)	2,908 (1,540–4,275; 63.2) 1,331 (916–1,746; 28.9) 365 (66–665; 7.9)	1,372 (804–1,939; 50.2) 1,093 (537–1,649; 40.0) 267 (2–531; 9.8)	0.33
Sex, n (95%CI; %)			
Female Male	1,309 (786–1,833; 28.4) 3,294 (2,074–4,515; 71.6)	1,312 (646–1,978; 48) 1,419 (810–2,029; 52)	**0.04**
Race, n (95%CI; %)			
White Black Other/unknown	2,454 (1,582–3,326; 53.3) 851 (1–1,701; 18.5) 1,299 (465–2,133; 28.2)	871 (466–1,275; 31.9) 582 (138–1,027; 21.3) 1,278 (574–1,983; 46.8)	**0.03**
Primary genitourinary organ injured, n (95%CI; %)			
Female external genitalia Scrotum/testis Penis Kidney Bladder Urethra Other	1,138 (648–1,628; 24.7) 1,139 (640–1,639; 24.7) 1,329 (448–2,209; 28.9) 602 (173–1,031; 13.1) 130 (0–280; 2.8) 115 (0–261; 2.5) 151 (0–341; 3.3)	1,102 (536–1,667; 40.3) 740 (194–1,285; 27.1) 496 (173–819; 18.2) 205 (0–420; 7.5) 5 (0–15; 0.2) 5 (0–15; 0.2) 180 (21–339; 6.6)	0.10
Diagnosis, n (95%CI; %)			
Laceration Contusion/abrasion Burn Fracture Other	1,023 (479–1,568; 22.2) 1,250 (555–1,945; 27.2) 55 (0–165; 1.2) 33 (0–99; 0.7) 2,242 (1,214–3,272; 48.7)	1,178 (562–1,795; 43.1) 430 (57–802; 15.7) 125 (0–252; 4.6) 132 (0–322; 4.8) 866 (439–1,292; 31.7)	**0.04**
Location, n (95%CI; %)			
Home Public Sports School Other/unknown	2,049 (917–3,181; 44.5) 314 (37–591; 6.8) 755 (353–1,156; 16.4) 320 (42–598; 7) 1,166 (425–1,907; 25.3)	1,629 (876–2,381; 59.6) 148 (0–341; 5.4) 0 0 954 (452–1,457; 34.9)	**0.03**
Disposition, n (95%CI; %)			
Treated/examined and released Hospitalized Left without being seen Died in ED	3,543 (2,313–4,773; 76.9) 844 (353–1,335; 18.3) 162 (0–488; 3.50 55 (0–165; 1.2)	2,393 (1,535–3,251; 87.6) 333 (81–586; 12.2) 5 (0–15; 0.2) 0	0.19

Values may not add due to rounding errors

^a^Statistically significant *p* values are shown in bold typefaces

CI, confidence interval; ED, emergency department; SE, standard error

Mean daily ED visits decreased significantly from the 2019 control period to the lockdown period in children ≤ 17 years (76.5 [95%CI 56.3–96.7] vs 36.1 [95%CI 19.0–53.2]; *p* < 0.01), males (86.7 [95%CI 65.4–108.0] vs 37.3 [95%CI 22.3–52.4]; *p* < 0.001), and White population (64.6 [95%CI 43.6–85.5] vs 22.9 [95%CI 12.4–33.4]; *p* < 0.01). Furthermore, injuries associated with sports (19.9 [95%CI 8.1–31.6] vs 0) and schools (8.4 [95%CI 0.8–16.1] vs 0) decreased significantly during the lockdown period. However, mean daily ED visits for injuries in adults between 18 and 64 years (35.0 [95%CI 20.0–50.0] vs 28.8 [95%CI 17.2–40.4]; *p* = 0.92), old adults ≥ 65 years (9.6 [95%CI 1.2–18.0] vs 7.0 [95%CI 0.1–13.9]; *p* = 0.37), females (34.5 [95%CI 21.5–47.4] vs 34.5 [95%CI 18.6–50.5]; *p* = 0.60), Black population (22.4 [95%CI 9.4–35.4] vs 15.3 [95%CI 5.5–25.1]; *p* = 0.90), and other/unknown races (34.2 [95%CI 18.5–49.9] vs 33.6 [95%CI 20.0–47.3]; *p* = 0.93) did not significantly decrease during the lockdown period. In addition, mean daily ED visits for injuries sustained at home (53.9 [95%CI 33.0–74.8] vs 42.9 [95%CI 27.2–58.5]; *p* = 0.75), public (8.3 [95%CI 0.6–15.9] vs 3.9 [95%CI 0–8.9]; *p* = 0.11), and other/unknown locations (30.7 [95%CI 17.1–44.3] vs 25.1 [95%CI 13.2–37.0]; *p* = 0.88) did not change significantly.

Table 2 demonstrates the top 10 consumer products with most changes in the number of ED visits for genitourinary injuries. During the lockdown period, injuries associated with toilets (− 320, − 74.6%), day wear (− 266, − 77.7%), beds/bedframes (− 209, − 64.2%) decreased while injuries associated with knickknacks/statues/vases (+ 154, n/a), sofas/couches/divans (+ 130, 2,684%), and razors/shavers (+ 99, n/a) increased.

**Table 2:** Consumer products with most changes in the number of traumas during the lockdown period compared to the control period in 2019

	Highest decrease in the number of ED visits during control (2019) versus lockdown period (2020)	Highest increase in the number of ED visits during control (2019) vs lockdown period (2020)
Consumer products, change in ED visits (n, Δ%)	1. Toilets: − 320 (429 vs 109, − 74.6%) 2. Day wear: − 266 (342 vs 76, − 77.7%) 3. Beds/bedframes: − 209 (325 vs 116, − 64.2%) 4. Bathtubs/showers: − 170 (259 vs 88, − 65.9%) 5. Bicycles: − 168 (430 vs 262, − 39.1%) 6. Playground climbing apparatus: − 155 (155 vs 0, − 100%) 7. Stairs/steps: − 120 (216 vs 97, − 55.2%) 8. Ladders: − 110 (110 vs 0, − 100%) 9. Basketball: − 109 (109 vs 0, − 100%) 10. Football: − 107 (185 vs 78, − 57.8%)	1. Knickknacks/Statues/Vases: + 154 (0 vs 154, n/a) 2. Sofas/Couches/Divans: + 130 (5 vs 134, 2684%) 3. Razors/Shavers: + 99 (0 vs 99, n/a) 4. Exercise (activity or apparel): + 78 (0 vs 78, n/a) 5. Scooters (unpowered): + 76 (0 vs 76, n/a) 6. Other clothing: + 76 (0 vs 76, n/a) 7. Refrigerators: + 76 (0 vs 76, n/a) 8. In-line skating: + 73 (5 vs 78, 1518.9%) 9. Desks/Chest/Buffets: + 72 (0 vs 72, n/a) 10. Toys: + 60 (16 vs 76, 386.2%)

Values may not add due to rounding errors

ED, emergency department

## Discussion

To our best knowledge, this is the first study to elaborate on changes in the pattern and characteristics of genitourinary trauma during the COVID-19 pandemic. We observed a significant decrease in ED visits for genitourinary traumas related to consumer products during the lockdown period compared to the identical period in 2019. However, no significant changes were observed for the periods between identification of COVID-19 and lockdown, and after reopening of businesses although it approached the significance level.

Genitourinary trauma contributes to trauma-related morbidity and mortality as it has reportedly been involved in up to 10% of patients presenting with trauma [11]. While the overall trauma admissions have been significantly impacted by the restrictions imposed due to the COVID-19 pandemic, the literature still lacks documentation on genitourinary traumas. Reports on trends of trauma during the pandemic have predominantly unveiled a decremental trend during the lockdown period. In a study on level 1 and 2 trauma centers in Los Angeles County, Ghafil et al. witnessed a significant decrease (incidence rate ratio = 0.92) in average weekly trauma admissions during the national lockdown compared to the identical period in 2019 [12]. A systematic review on 57 studies from the United Kingdom, Europe, Asia, Australia, New Zealand, and the US underlined a global decrease (20.3–84.6%) in trauma cases compared to the pre-pandemic years [8]. As indicated by our analysis, consistent with other types of trauma, genitourinary traumas exhibited a similar decremental trend during lockdown compared to the control period in 2019. Such decrease and shift in the patterns of genitourinary trauma potentially stem from the limitations in social interactions imposed by the execution of stay-at-home orders. Additionally, patients’ hesitation in visiting EDs motivated by the fear of contamination, not wanting to burden health care system, perceiving own complaints less urgent relative to COVID-19 patients, and limited access to services may also reduce trauma presentations [13, 14].

Despite trauma studies predominantly indicating an increased proportion of male trauma presentations during the COVID-19 pandemic, our results exhibited a significant drop of more than half in men’s daily ED visits due to genitourinary traumas. On the other hand, we found that the product associated with the highest decrease in ED visits during the lockdown period was toilets. A previous study indicated that the most common mechanism associated with toilet- and toilet seat-related genitourinary injuries was crush from accidental fall of toilet seat (68.4%) [15]. In addition, most crush injuries were isolated to the penis (98.1%) and younger children. We similarly observed a significant drop in penile injuries and injuries in children which mirrors the decrease in toilet-related genitourinary injuries. The reason for this observation could be the use of standard slow-close toilet seats at home, in addition to being in a familiar environment under parental supervision.

Several reports have indicated an increased rate of lacerations in trauma patients during the pandemic [16]. Similarly, our results demonstrated a surge in the ED presentations with genitourinary laceration as the diagnosis. The change in trauma patterns in favor of house items and grooming products could explain this finding. Moreover, the increase in the proportion of genital burns could be attributable to more frequent use of the motorized products along with the increase in the use of fireworks, flares and fuel-burning equipment [17].

The COVID-19 pandemic has changed the lifestyle and leisure time habits among adults and children. School closures have led to deprivation from social interactions in children, and public sports were suspended likewise. Our analysis revealed a considerable increase in home versus public genitourinary injuries. Sports are known as a significant contributor to contusions and abrasions in traumatology [18, 19]. In our study, no cases of genitourinary injury secondary to sport injuries were estimated during the lockdown period. Furthermore, we noticed a significant decrease in the proportion of genitourinary trauma patients diagnosed with contusions and abrasions. Interestingly, fewer patients with genitourinary traumas were hospitalized and fewer died of their injuries during the lockdown period. This may reflect a lower incidence of severe poly-trauma (including those with genitourinary injury) or a trend toward less severe trauma during the lock-down period.

This study has some limitations that should be acknowledged. The NEISS only reports non-violent trauma cases referred to the US EDs and minor injuries treated without a hospital visit are not included in this database. As such, this could underestimate the actual number of genitourinary injuries. In addition, due to a relatively low number of actual cases in some subgroups of the study, the national estimations could be unstable. Despite such limitations, this study provided novel insights on genitourinary traumas during this critical time.

## Conclusions

Genitourinary traumas have exhibited a decremental trend during the COVID-19 lockdown period compared to the pre-pandemic control period. There was a significant shift in patient and injury characteristics. Average daily ED visits in children < 17 years, males, and White population decreased significantly although it remained unchanged in females. Similarly, penile injuries which was the most common organ injured before the pandemic, dropped significantly during the lockdown period. In addition, the pandemic and stay-at-home orders were associated with less severe genitourinary traumas. These could be beneficial as hospitals and healthcare institutions deal with a surge in the number of COVID-19 patients. As such, the contributing factors to the observed changes in the patterns of genitourinary trauma presentations could be investigated further to minimize these types of injuries during post-COVID-19 normalcy.

## Data Availability

The NEISS data is publicly available.
